# Deterioration and Protection of Concrete Elements Embedded in Contaminated Soil: A Review

**DOI:** 10.3390/ma14123253

**Published:** 2021-06-12

**Authors:** Ginneth Patricia Millán Ramírez, Hubert Byliński, Maciej Niedostatkiewicz

**Affiliations:** Department of Concrete Structure, Faculty of Civil and Environmental Engineering, Gdansk University of Technology, 80-233 Gdańsk, Poland; mniedost@pg.edu.pl

**Keywords:** concrete corrosion, concrete protection, steel corrosion, concrete durability, coating materials

## Abstract

Coating materials are considered one of the most antique materials of human civilization; they have been used for decoration and the protection of surfaces for millennia. Concrete structures—due to their permanent exposure to different types of environments and contaminants—require the use of coatings that contribute to its preservation by reducing the corrosion of its components (steel and aggregates). This article intends to introduce the principal causes of concrete deterioration and the coating materials used to protect concrete structures, including a summary of the coating types, their advantages and disadvantages, and the latest developments and applications. Furthermore, this paper also assesses brief information about the potential challenges in the production of eco-friendly coating materials.

## 1. Introduction

In the last few decades, reinforced concrete (RC) has become one of the most used construction materials. Its versatility and adaptability offer infinite applications in the construction sector [1,2]. The construction industry has been looking for several methods to improve the durability of concrete structures; rehabilitation, restoration, and strengthening are the most common activities to extend an existing structure’s life cycle [3]. The durability of concrete structures embedded in soil and exposed to different types of contamination might be affected by two factors: deterioration from concrete components and chemical deterioration caused by external agents [4,5]. Table 1 summarizes the factors involved in the decrease of the durability of structures exposed to contamination.

Construction, energy, mining, agriculture, and transport industries, are one of the primary sources of contaminants; according to Enshassi et al. and Zolfagharian et al. [10,11], these can be defined as solid and liquid waste, harmful gases, noise, water, soil, and air pollution. Even though the construction sector causes several impacts to the environment, this sector is also affected by the pollutants released by other industries, e.g., soil contamination due to agricultural and mining activities reducing the durability of structures embedded in the soil caused by the presence of chemical compounds, and air pollution produced by energy and transport sectors, where the emanation of chlorine oxides contributes to the accelerated corrosion [12,13]. For this reason, it is essential to develop processes that generate less contamination and allows the protection of construction elements exposed to contaminants.

Previous studies have focused on the durability, deterioration, and service life of concrete structures, including numerical models [14,15,16] and experimental studies [17,18]; however, these studies mainly focused on constructions located above ground level and ignored the impact of the different factors on the structures located below ground level. Wei et al. [19] investigated how acids coming from the atmosphere and retained in the superficial layers of the ground induce concrete degradation decreasing the compressive strength and increasing the corrosion coefficient of concrete; it was identified that the main reason for premature deterioration of concrete is due to the changes in temperature where the corrosion coefficient was increased about two times for samples exposed to 40 °C. However, the compressive strength results did not show any significant changes during the 90 days of exposition. Kozubal et al. [20] have proposed a numerical model that allows preventing structural damage of vertical elements exposed to a contaminated soil environment. This model permits design engineers in the decision-making process by ensuring the safety of concrete structures embedded in the soil. The mathematical model was proposed based on the deterioration of concrete Controlled Modulus Columns (CMC) exposed to different sediments in groundwater, evidencing the apparition of cracks due to chemical corrosion. Li et al. [21] presented an analytical approach to predicting the life span of reinforced concrete pipe piles that are constantly exposed to chloride contamination and are affected by the earth pressure causing deterioration of the elements by the diffusion of microcracking. Among the principal assumptions, it can be highlighted that the end of the service life of these structures is going to be reached once the elements present total transverse cracks allowing the penetration of chlorides into the concrete core; this method provides a genuine approach for the evaluation of service life of concrete pipe piles allowing the improvements of durability design and reducing the maintenance of this concrete elements.

**Figure 1:**
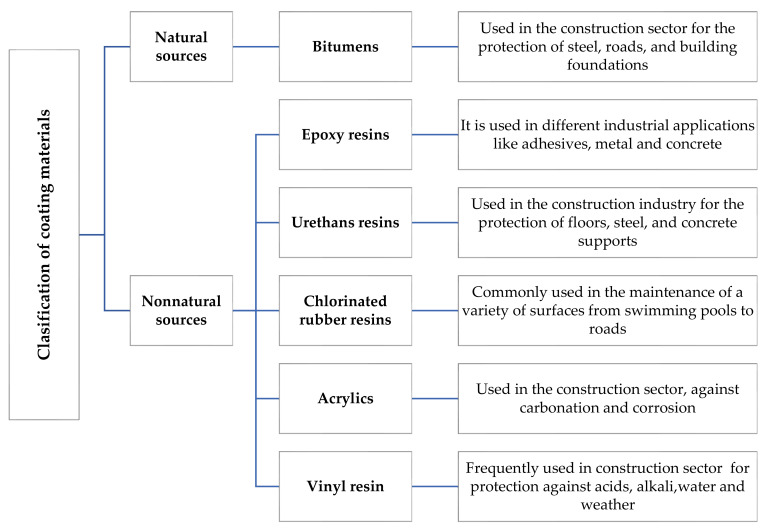
General classification of coating materials used in different industries [24,25,26,27,28,29,30,31,32,33].

Recently, different coating materials have been used to protect concrete structures in the construction industry. Among the most common ones, it is possible to find fire protection coatings used as a precautionary measure preventing buildings from collapsing during fire exposure [22] and waterproof coatings widely used in the protection of concrete against reinforcement corrosion, erosion, carbonation, silica reactivity in aggregates, and chemical attacks, such as acids, salts, alkalis, and sulfates [2,7,23]. The use of coatings also increases the structure’s lifetime by preventing the appearance of cracks and reducing the maintenance cost. Figure 1 shows the general classification of coating materials for different industries.

In the last few decades, research studies about the utilization of coating materials as protection for concrete elements exposed to different environments have increased due to the significant growth of this sector and the development of a large diversity of coating materials, varying not just raw ingredients but also the process of manufacture; among the most common techniques for the preparation of coating materials, it is possible to distinguish the solution casting method proposed by Sakamaki [34], the phase-transfer catalyst process, the taffy process, and the fusion process [30]. Table 2 summarizes the historical milestones in the development of coating materials from prehistory until the present day.

Generally, coating materials are commonly used in concrete structures when they are exposed to contaminants. Zouboulis et al. [39] proposed the study of corrosion protection of concrete samples covered with six different coatings with magnesium hydroxide against contaminants contained in sewage systems. This study has been developed in a controlled environment in a laboratory simulating the biological contamination produced in an actual sewage plant using a sulfuric acid solution and using concrete type MC 0.45 simulating the concrete used in the sewage pipes, the grade of protection of the coating was evaluated with an accelerated degradation method by spraying $H_{2}SO_{4}$ in the surface sample, this process was performed until the coating’s degradation was evidenced visually. Among the results, it is possible to identify that the thick layer of the coating material is directly related to the durability time, samples with $0.002\;g/mm^{2}$ presented double duration time than the samples covered with $0.001\;g/mm^{2}$, also the XRD analysis showed that all samples obtained gypsum formations before the total degradation of the coating material, even though the coating material presented degradation, its superficial pH was constant in all cases, maintaining an average value slightly over 8. Aguirre-Guerrero et al. [40] evaluated the protection effectiveness of inorganic coatings applied to concrete exposed to chloride contamination by analyzing different properties, such as water absorption, resistance to chloride ion penetration, adhesion strength, and corrosion resistance. Among the results, it is important to mention that coated concrete has not performed well, presenting lower resistance to water penetration and an increment in their capillary absorption. However, all concrete samples protected with inorganic coating showed an increment in chloride penetration resistance compared to concrete samples without protection by reducing the penetration of chlorides from high to moderate and, in some cases, to low. Finally, the use of coatings prolongs corrosion and extends the time of cracking. Sakr et al. [41] studied how different coating materials protect concrete with different water–binder (w/b) ratios when exposed to constant salt attack. It is evidenced that acrylic emulsion, epoxy, and ethyl silicate successfully protect concrete surface from physical salt attack regardless of the type of concrete and salt concentration. At the same time, the protection capacity of coatings made with the addition of fly ash strongly depends on the concrete (w/b) ratio. In general, coating materials successfully protect concrete against different types of chemical aggressions extending the lifespan of concrete elements and reducing the maintenance of structures.

This review paper aims to review the most relevant and recent investigations related to the use of coatings materials for the protection of concrete exposed to different types of contamination, also it reviews the deterioration of concrete exposed to a contaminated environment by summarizing the relevant manuscripts published in the last five years, until 2021. Table 3 and Table 4 shows the statistical data of the resources used in this review paper, such as total of publications used per year and per country. The research gaps in the implementation of coatings materials and challenges for the future are identified and discussed.

## 2. Search Methodology

This study’s research adopted the steps proposed by Ferenhof and Fernandez [42] for the systematic search flow method (SSF) to obtain the necessary information to develop this paper. The SSF method consists of four core steps:

**i.**  **Search protocol:** A set of rules and parameters for the search process was used together with logical and relational operators (AND, OR, NOT, <, >, <=, >=, < >, =, etc.).

*Keywords:* Concrete, corrosion, concrete protection, durability, concrete degradation, carbonation depth, alkali reaction, diagnosis, repair, steel corrosion, chemical attack, soil contamination, coating materials, organic coating, non-organic coating

*Databases:* MDPI, SpringerLink, Elsevier—Science Direct, Scopus, Access Engineering, ASTM (American Society for Testing and Materials)

*Year of publication:* 2010–2021 for study cases

**ii.**  **Analysis:** It refers to the consolidation and combination of data according to different criteria, such as most-cited authors, year of publication, and type of journal by creating a database with various articles that meet the search and consolidation criteria.

A database was developed using an online tool containing basic information of the articles selected, such as author name, title, year of publication, journal of publication, organized by the main topic: coating materials, soil contamination, concrete degradation, and steel degradation.

**iii.**  **Data synthesis:** It allows to generate conclusions and new knowledge based on the results presented by the different papers analyzed.

The database prepared in the analysis section was extracted to a spreadsheet and evaluated, resulting in selecting the papers to be used.

*Article selection:* 210 articles were selected, 162 articles were read, and 76 articles are referenced in this paper

**iv.**  **Writing:** The information was extracted from 76 articles. The results were consolidated through scientific and academic writing.

## 3. Significance of the Review

The principal purpose of this research paper is to contribute to the comprehensive state-of-the-art about the corrosion of concrete elements that are embedded in contaminated and noncontaminated soils, together with a brief overview of the current coating materials used in the construction sector. This paper summarizes all relevant data from different articles, such as types of laboratories, exposition time, sample size, etc., and determines the principal causes and consequences of contamination.

From the analysis of the articles, it is possible to determine that the main cause of corrosion in elements exposed to contaminated soil is the contamination generated by human activities, construction, mining, agriculture, and others. On the other hand, it is possible to state that there is no evidence regarding the use of coating materials to protect concrete elements located below ground level, representing a wide area of research with high potential.

## 4. Results and Discussion

### 4.1. Chemical Corrosion of Concrete Elements in Contaminated and Noncontaminated Soil

**Figure 2:**
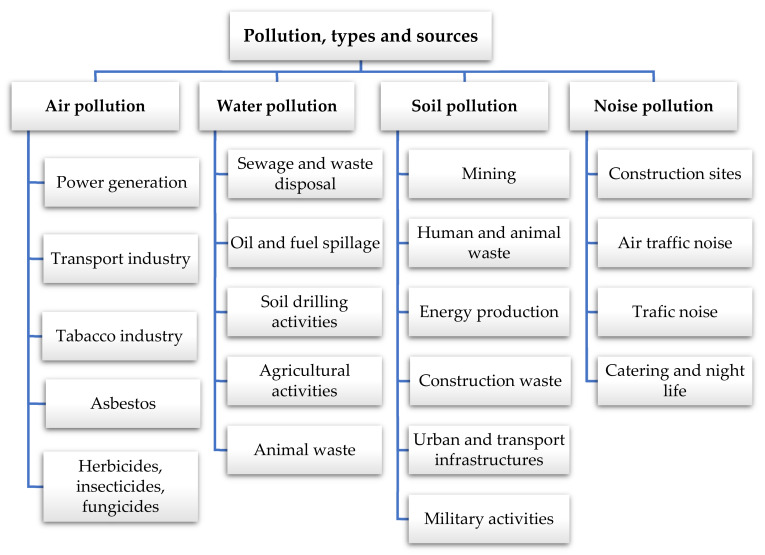
Main sources of soil pollution [10,43,45,47,48,49].

During the last few decades, the continuous growth of the human population has contributed towards increasing different industrial activities, such as agriculture, energy, transport, construction, technology, and mining. These, in turn, increase soil pollution [43], the loss of crop diversity, productivity, and soil quality by decreasing its mechanical and physical properties, such as electrical conductivity, bulk density, pH, moisture content, and hardness [43,44]. Figure 2 summarizes the main types and sources of soil pollution.

Heavy metal contamination is one of the most severe types of contamination; uranium, arsenic, cadmium, tin, lead, manganese, vanadium, and mercury are the most abundant metallic pollutants introduced into soil through the use of fertilizers and pesticides in the agriculture industry. Human exposure to these metals can lead to several body dysfunctionalities and damage, including depression, osteoporosis, liver disease, and anemia [45]. Coal-fired and nuclear power plants are the primary producers of $CO_{x},NO_{x},\;SO_{x},\;UO_{x}\;$ and some radionuclides contaminants such as, ${\;}^{137}Cs_{\;,}{\;}^{134}Cs_{\;}$, which are deposited into the soil by deposition (fallout) or by precipitation after being dissolved in the rain, contributing to global warming, acidification increase, depletion of the ozone layer, health problems, and soil contamination [46]. Finally, mining, agricultural, construction, and transport industries are the principal generators of petroleum hydrocarbons contamination, spilling different types of fuel and oils into the soil. Extraction of metals and minerals can carry chemicals and metals that may contaminate water bodies located nearby and potentially affecting human and wildlife health [44].

### 4.2. Characteristics of Reinforced Concrete Elements Embedded in the Ground in Terms of Their Chemical Corrosion

Concrete structures that are located below the level of the ground are exposed to different types of contamination. Some of them come from natural sources; however, most of them are related to human and industrial activity [47,48]. Other types of research have been conducted to determine the impact of soil contamination on foundation structures. Table 5 summarizes the most common laboratories performed in the articles included in the methodological search.

One of the most concerning topics about cement-based structures is their durability when exposed to different chemically aggressive scenarios causing its degradation; these scenarios can be classified into three groups: physical, biological, and chemical [12], which can be contained in contaminated soil and water [12,50]. There are different methods used to determine the resistance of samples formed in cement paste, mortar, or concrete; these might vary in the type of exposure, sulfate concentration, and temperature, where expansion behavior, relative flexural strength, compressive strength, permeability, and elastic modulus are the most common measurements tested in concrete and mortar samples to determine the deterioration caused by the exposition to different contaminants. [51].

Osuji et al. [52] analyzed the reduction in compressive strength of concrete samples with fine and coarse aggregates contaminated with crude oil and its influence on concrete workability. The slump test evidenced that the inclusion of contaminated aggregates impacts the workability of the fresh concrete, increasing the slump results from 45 mm to 165 mm, which leads to segregation and prevents the correct hydration of cement. The compressive strength result showed a reduction of about 64% compared to the control test due to the segregation of the materials evidenced in the slum test; based on this, it is suggested to avoid the use of fine and coarse contaminated aggregates in mixtures.

In the study conducted by Adewuyi et al. [53,54], concrete samples of different dimensions were exposed for 215 days to biological contamination caused by organic abattoir waste and diesel and cassava hydro-cyanide contaminated soil. The results indicate that aggressive environments attack the concrete’s physical and mechanical properties, leading to a reduction in the compressive strength of about 10% in the samples exposed to the cassava-contaminated soil. The specimens in the abattoir waste were additionally exposed to progressive heat, up to five temperature cycles to accelerate its degradation. The final results show that exposure to hydrocarbon (diesel) contamination is more severe on concrete samples than the organic contamination caused by the abattoir; samples exposed to diesel presented a reduction in their compressive strength of around 22–28% against 12–20% for samples exposed to abattoir contamination, in both cases, this reduction is caused by the loss of porosity and the decrease of mass which was higher in the specimens exposed for a longer time.

Yu et al. [55] exposed cylindrical and prismatic mortar samples for 270 days to $Na_{2}SO_{4}$ solution, the samples were also subjected to dry-wetting cycles with 0% and 5% of the solution to determine its compressive strength, elastic modulus, permeability, and expansion behavior. Results showed that the maximum expansion obtained was approximately 0.6%, being 0.5% higher than the expansion limit stated in the ASTM C1012-2014. On the other hand, compressive strength results performed at exposure durations up to 270 days showed a reduction in the resistance of about 30% in the samples due to the microcracking caused by the dry-wetting cycles and the deterioration of the material due to the constant exposition to sulfate solution. It was possible to evidence an increment from 14.6 GPa to 18.0 GPa in the elastic modulus during the first 150 days of exposure and then this decreased to around 14.0 GPa at 270 days. All samples exposed to a variation in temperature and sulfates exhibited a deterioration at a larger stage that affects the material quality and durability along with the accumulative microcracking.

Carbonation is also known as a major cause of deterioration of concrete structures embedded in contaminated soil, this type of corrosion depends on different factors, such as $CO_{2}$ pollution, water, temperature, curing process, W/C ratio, and the characteristics of the materials that compose the concrete. It is a pathology of the reinforced concrete that causes reinforcement depassivation, exposing the steel to corrosion, and its development is highly influenced by the different environmental and exposure conditions. Destructive and non-destructive tests are used to diagnose the degradation of concrete samples due to carbonation, such as visual inspection of samples, determination of the reinforcement coating, measurement of compressive strength and concrete cover, and measure of carbonation [56].

A phenolphthalein indicator is commonly used to determine the carbonation depth, being sprayed onto the surface of a freshly cut sample. Chang et al. [57] shows the results of twenty-four cylindrical models made with ordinary Portland cement and subjected to an accelerated carbonation process in a chamber at 23 °C, 70% relative humidity, and 20% of $CO_{2}$ concentration during 8 and 16 weeks. The average carbonation depth for the phenolphthalein solution was about 12 mm for the specimens exposed for 8 weeks and 17 mm for the samples exposed for 16 weeks, this led to a change in the pH of the concrete from 9.0 to 7.5, where the degree of carbonation reached 100%.

Foundation structures are exposed continuously to different aggressive agents, such as chlorides and sulfates during their service lifespans [58,59]. Chloride ions are present in industrial water, seawater, contaminated soils, and sewage water ions [60], the exposure to these is the main cause of corrosion of reinforced concrete structures and one of the most critical problems of structures embedded in the ground. Particularly, the steel bars of concrete structures can be corroded by these chemical agents present in soil, thus affecting the structure’s durability.

By the measurement of potential and velocity of corrosion, Baltazar-Zamora et al. [58] observed that the carbon and galvanized steel used in concrete samples exposed to soil contamination with sodium chloride content higher than 2% for 257 days presented a very high probability of suffering from premature corrosion; however, the compressive strength of the different samples was not compromised, since none of them showed a reduction in their mechanical properties.

Table 6 and Table 7 present a summary of the exposition times of concrete samples to contaminated environments and their size characteristics, respectively.

### 4.3. Characteristics of Emergency State of Structures Caused by Chemical Corrosion of Concrete Elements Embedded in the Ground

Concrete structures are exposed to constant environmental impacts that affect their physical and mechanical properties [10]. In constructions that are located above ground level, it is easy to determine damages and the level of impact on reinforced concrete due to different contaminants or construction and structural design errors. However, concrete structures below the ground are impacted more severely due to the constant exposure, lack of supervision, and preventive maintenance, resulting in damages that would be difficult to identify and repair. Hence, these damages can potentially affect the bearing capacity and durability of the structures mentioned above [12]. The following research presents real-life examples of structures exposed to different types of contamination where the causes and consequences of constant exposure are known and presented.

Zhong et al. [61] analyzed the premature corrosion of concrete foundations in residential buildings located in Eastern Connecticut in the United States; this deterioration is related to the expansion of the aggregate, caused by the alkali–silicate reaction (ASR) and internal sulfates attacks, resulting in map cracking and wide crack openings in foundation elements [62]. To determine the original causes of the aggregate expansion, 70 core samples were taken from different residential house foundations affected by premature corrosion. Compressive strength results show that 30% of the samples obtained 0 MPa due to the high deterioration level, falling apart even before the test was done, and 20% of the specimens had a strength reduction of about 57%. From the X-ray diffraction (XRD) tests and the use of scanning electron microscopy, it was possible to determine that the samples with the highest deterioration level had a significant content of sulfide iron mineral in the form of pyrrhotite, which was found to be responsible for the premature concrete deterioration by oxidation, which facilitates the formation of secondary minerals that release sulfates.

Similar results were found in the research conducted by Tagnit-Hamou et al. [63], where building foundations in Eastern Canada presented several deterioration problems two years after the construction. Different cores were taken from the foundations to check the causes of corrosion, and according to the XRD results, the cement matrix and aggregates were affected by the presence of pyrrhotite, causing the early cracking of the concrete.

Another example of the deterioration of concrete foundations is given by Yoshida et al. [64], where residential buildings in Japan were affected by sulfate attacks; this is considered an important problem for hot springs and mining areas. According to the Japanese Geotechnical Society, soil samples were checked to evaluate the sulfate content, where the values of water-soluble sulfate exceeded the standard’s criteria, reaching, in some cases, more than 1.0% of the mass soil. In addition, small concrete cores were taken from the deteriorated foundations of residential buildings. It was evidenced in these samples that the penetration of sulfur trioxide was around 20 mm. This type of sulfate attack was classified as a “physical attack” due to the minimum cracking on the element’s surface.

Other types of sulfate attack in concrete foundations can be found in sewage water, which leads to the degradation of the elements due to sulfuric acid produced by the different microorganisms present in the contaminated water, reducing by this, mechanical properties of the concrete and the loss of adhesion of the cement matrix. Tulliani et al. [65] evidenced in their research a severe degradation case in a 35-year-old building located in the north of Italy, where concrete samples were taken from the foundation elements and analyzed by X-ray diffraction (XRD) and scanning electron microscopy (SEM). It was evidenced that the bond between the coarse aggregates and the cement past was poor, and also that the steel reinforcement was highly corroded. For samples without corrosion, the pH and conductivity presented values of 7.5 and 305 µS, respectively; however, for the specimens with severe damage, the pH and electrical conductivity were about 7.2 and 1650 µS. SEM and XRD analyses showed a high gypsum concentration between cement and aggregates responsible for strength loss.

Based on previous research, it is evidenced that the presence of different minerals and contaminants produce chemical reactions that lead, in some cases, to severe corrosion and thus degradation of the elements embedded in contaminated soil, which results in the effects on their mechanical and physical properties.

Table 8 contains a summary of the most interesting study cases related to those evaluated in Section 4.2 and Section 4.3.

### 4.4. Coating Materials—Current State, Challenges, and Perspectives

#### 4.4.1. Research Gaps in the Use of Coating Materials

The rapid and continuous growth of different industries, the lack of control in the production of materials, food, and poor waste management, can eventually increase air and soil pollution, as evidenced in previous chapters, decreasing the service life of structures exposed continuously. Different materials have been implemented over the years to protect concrete elements by reducing corrosion at an early age. As Table 6 demonstrates, most of the researches are focused on analyzing the concrete and steel mechanical properties behavior in structures located above the ground when exposed to different types of contamination, either organic and non-organic. However, the use of coating materials is not evidenced for the protection of concrete elements embedded in the soil, taking this into account, it is crucial to invest in the research of coating materials that can be applied in concrete elements embedded in contaminated soil that allow the preservation of structures exposed to different types of contamination at various degrees.

#### 4.4.2. Current Status and Future Challenges

**Figure 3:**
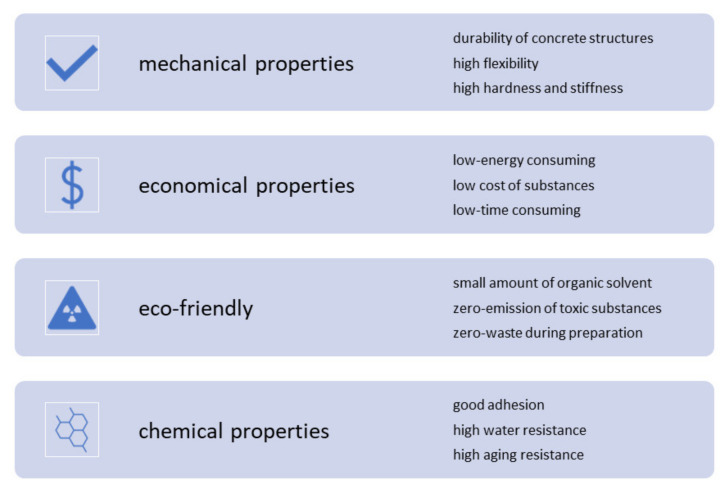
Critical aspects in the field of challenges of production of coating materials used for protection of concrete elements.

Protective coatings are present in most of the surfaces around us, used from the simple protection of food to the complex protection of steel and concrete. Nevertheless, most of these coatings go through a manufacturing process that generates contamination. Some of them use nonrenewable materials, such as bitumen obtained from petroleum refining, causing several environmental problems. In addition, some of the coatings use organic solvents that emit volatile organic compounds, producing air pollution that affect human health. Therefore, it is necessary to continue developing eco-friendly coating materials that contribute to environmental preservation without sacrificing the main properties of the materials, e.g., high durability, toughness, adhesion, strength, etc. Table 9 contains the main advantages and disadvantages of some of the most common coating materials used in the construction industry to protect concrete structures. Figure 3 shows the key aspects and challenges in the production of coating materials.

#### 4.4.3. Characteristics of Coating Materials According to Polish–European and American Standards

Coating materials must follow the specifications stated in the European Standards (Eurocode) regarding coating adhesion to the substrate, absorption, and permeability, among others. Table 10 contains the most imperative standards describing the physical and mechanical properties of coating materials intended to protect different surfaces, such as wood, steel, concrete, plastic, and glass. For this paper, the PN–EN standards based on the European Standards will be taken as a reference.

Table 11 summarizes the laboratories that performed evaluations of the physical and mechanical properties of coating materials used in the construction industry according to the academic articles used in the search methodology.

Approximately 54% of the articles reviewed applied Polish–European standards (PN-EN), 38% used the American Society for Testing and Materials (ASTM), and just 2% used local test methods approved by the ITB (Building Research Institute) in Poland. The most common procedures among the literature were adhesion, tensile stress, and resistance to freeze/thaw cycles tests. In addition, the article analyses focused on the reduction and control of the carbonation process and on the proposal of new coating materials for the protection of concrete exposed to contaminated environments.

During the last decades, different raw materials have been used to produce new protective coatings materials intended to improve the concrete properties. Elnaggar et al. [61] presented a novel protective material based on different ratios of isocyanate chemical groups (NCO) and a mix of 80% asphalt and 20% polyester. The asphaltic polyurethane (As/PU) coating was tested on concrete cubes; according to the results, an increment in the dry film thickness was shown, from 86µm to 98µm, in samples with a 1:4 ratio of NCO, which can be attributed to the density of the (As/PU) coating. Similarly, adhesion strength showed an increase of 145% in the samples with a 1:4 ratio of NCO, an effect that can occur due to the interaction between ACO groups and ANH. Finally, it was concluded that both dry film thickness and adhesion strength improved with the increase in NCO/OH ratio.

Francke et al. [62] proposed a new coating material modified with cementitious mortar to perform waterproof and chemical protection. Based on polymer–cement products, this coating material effectively performed the functions of concrete carbonation protection by reducing the carbonation depth by 24% and increasing in 7% the adhesion strength in frost and storm environments. However, in freeze–thaw cycles with the addition of sodium chloride solution (salt), a decrease was evidenced in the bonding strength of about 40% with respect to the sample without environmental exposure.

Improving the protection of concrete structures is one of the most critical objectives in manufacturing new coating materials. Significant results have been evidenced by applying protective (As/PU) layers showing a reduction in the chloride penetration of about 75% with respect to the control sample. Even though the immersion of both coated and non-coated samples in sulfuric acid and NaCl solutions show a decrease in the compressive strength of the concrete samples, it can be evidenced that the coated samples present a reduction in the compressive strength between 22% and 27% and a decrease of 50% in the non-coated material. Finally, it can be concluded that the protection with asphaltic polyurethane (As/PU) coating improves the mechanical and physical properties gradually when the ratio is increased with respect to the samples without coating.

According to Baba et al. [63], to minimize the corrosion caused by carbonation, concrete surface protection can be performed with three different coating materials: penetrants for surface improvement, non-cementitious for finishing layer, and cementitious for finishing layers. In the research conducted by Lo et al. [64], eight non-cementitious coatings, emulsions, and synthetic paints were used in concrete prisms to analyze their impacts on the reduction of carbonation depth; four of them were tested for interiors and the rest of the coatings for the exterior. In addition, an accelerated carbonation test method was implemented, exposing the samples to a constant $CO_{2}$ flow in a chamber for 56 days, the deep carbonation was measured by exposing the samples to phenolphthalein solution. Results showed that for exteriors coatings, the C25 concrete samples obtained a reduction in deep carbonation of about 60% and 45% for interiors coatings, decreasing from 16.40 mm to 6.58 mm and 8.93 mm, respectively. For C35 concrete samples a reduction in the deep carbonation for exteriors coatings of 56% (3.78 mm) was also evidenced, and for the interior coating it was 40% (4.23 mm). Based on this it can be concluded that there is a significant reduction in the corrosion caused by carbonation using these coating materials.

The authors mentioned above evaluated the benefits of coating materials in structures exposed to contamination. It was evidenced that coating materials effectively reduce the impact caused by different chemical attacks, and are able to extend the lifespan of concrete structures and reduce corrective maintenance costs.

## 5. Conclusions

This literature review was prepared to give an overview of the causes of corrosion of concrete elements exposed to different types of contaminants and the procedures proposed and used by some researchers to protect these elements. Different coating materials have been proposed, varying from naturals sources, such as bituminous coatings, to synthetic productions, like acrylic coatings. Among the results, in all cases where concrete samples were subjected to contamination either by exposition to chemical or natural contaminants, the compressive and flexural strengths showed a significant reduction. In addition, galvanized and carbon steel bars embedded in concrete samples showed an increase in corrosion, potentially leading to a premature corrosion of the bars and premature cracking and deterioration of the concrete elements. Even though several investigations have been carried out on how different types of contamination affect concrete, there is not much evidence yet on how coating materials can protect concrete elements embedded in contaminated soils.

## 6. Research Limitations

This review paper was limited to Spanish and English articles found in the journals mentioned in Section 2, “Search Methodology”, which excludes literature published in other languages and was limited to academic publications. It does not consider the results from industrial practice.

## Figures and Tables

**Table 1:** Summary of causes of deterioration of concrete structures exposed to contaminated soil [6,7,8,9].

Causes of Deterioration	Deterioration Type
**Caused by concrete components**	***Alkali–silica reaction (ASR)***: It is one of the most concerning topics regarding the durability of concrete, leading to costly maintenance and rehabilitation works. ASR occurs when cement aggregates react with the alkali hydroxides in concrete, producing a hygroscopic gel that in the presence of water causes an expansion and thus the cracking of the concrete surface ***Corrosion of steel bars***: The corroded bars occupy a greater volume than the non-corroded ones, causing cracking and delamination of the concrete surface. Steel corrosion is caused by the presence of chloride ions or carbon dioxide.
**Caused by external agents**	***Chemical corrosion***: It can be divided into two groups: i. *Chemicals that promote a rapid deterioration*: Aluminum chloride, calcium bisulfite, hydrochloric acid, nitric acid, and sulfuric acid. ii. *Chemicals that produce a moderate deterioration:* aluminum sulfate, ammonium bisul-fate, ammonium nitrate, ammonium sulfate, ammonium sulfide, and sodium bisulfate. ***Volume changes***: Freeze–thaw cycles, plastic and drying shrinkage, and thermal changes are the leading causes of volumetric change.

**Table 2:** Milestone chronology in coating history [35,36,37,38].

Year/Period	Event Description
**Pre-History**	
**Before 4000 B.C**	Varnishes and paints were used during the stone age art
**Before 6000 BC**	Development of organic pigments (gum Arabic, egg white, gelatin, and beeswax)
**Since 5000 BC**	Use of protective coatings by Egyptians to seal ships
**Ancient Age**	
**1300 B.C-1400 B.C**	Use of oleo-resinous varnishes by Egyptians
**Since 1122 BC**	Introduction of polymers as the main component in coatings
**350 B.C**	First written record of uses of varnishes
**Middle Age**	
**476 B.C-1453**	Use of different organic paints and varnishes for the protection of exposed wood surfaces
**Modern Age**	
**1550–1750**	Researches about coatings for protection of musical instruments made in wood
**1575**	The first use of yellow amber resin as a primary component in coatings
**Since 1760**	Significant emergence of coating materials as a high technology industry, the development of synthetic resins in solutions, emulsion, latexes, and waterborne polymers
**1763**	First varnish patent
**Contemporary Age**	
**1815**	Start industrial varnish production
**1839**	The first production of styrene monomer used as a modifier in polymer coatings
**1910**	Casein powder paints
**1912**	Patented acrylic resin
**1939–1945**	Development of alkyds, urethane, and epoxy resins
**1948**	Incorporation of latex resins in the coating industry
**1961–1965**	Development of coil coatings, electrodeposition curtain coating, computer color control electrostatic powder spray, fluorocarbon resins
**1970**	Use of emulsion resin to control penetration in substrates
**1966–1970**	Development of radiation curable coatings
**1970–1975**	Development of aqueous industrial enamels electron beam curing, and ultraviolet curing
**1976–1980**	Development of high solid epoxy and polyurethane coatings resins
**1981–1985**	Development of high-performance pigments, polyurea resins, and high solids alkyd paints
**1986–1999**	Waterborne epoxy coatings and waterborne polyurethanes
**21st Century**	New systems based on alkyd technology, synthetic polymer-based coating resins, e.g., PVC-plastisol, acrylate dispersion, melamine/polyester, 2K urethanes, and inclusion of new drier systems for alkyds by replacing the cobalt driers

**Table 3:** Total of documents per year.

Year	Total of Publications Used
**2021**	4
**2020**	3
**2019**	13
**2018**	6
**2017**	7
**2016**	6
**2015**	9
**2014**	3
**2013**	2
**2012**	4
**2010**	3
**2007**	2
**2005**	3
**2004**	1
**2002**	2
**2001**	2
**2000**	4
**1989**	3
**1983**	1
**1981**	1
**1978**	1

**Table 4:** Total of documents per country.

Country	Total of Publications Used
**USA**	20
**China**	13
**India**	7
**Germany**	6
**Nigeria**	4
**United Kingdom**	4
**Mexico**	3
**Poland**	3
**Canada**	2
**Spain**	2
**Italy**	2
**Portugal**	2
**Saudi Arabia**	2
**Australia**	1
**Brazil**	1
**Colombia**	1
**Czech Republic**	1
**Greece**	1
**Japan**	1
**Lithuania**	1
**Russia**	1
**Serbia**	1

**Table 5:** Laboratories described in the different articles selected in the methodology search.

Material	Laboratory Type	Number of Articles
**Concrete**	Compressive strength	19
	Flexural strength	4
	Loss of concrete weight	5
	Slump test	6
	Permeability	4
	Expansion behavior	4
	Carbonation depth	14
**Steel**	Corrosion potential	5
	Corrosion kinetics	5
**Aggregates**	Moisture	3
	Bulk density and gravity	3

**Table 6:** Summary of exposition time to contaminated environments according to the articles selected in the methodology search.

Exposition Time (Days)	Number of Articles
**<100**	4
**>100 and <200**	7
**>200**	13

**Table 7:** Summary of sample sizes according to the articles selected in the methodology search.

Shape	Sample Size (mm)	Number of Articles
**Prismatic**	120 × 150 × 70	5
	150 × 150 × 150	12
	150 × 100 × 1000	4
**Cylindric**	150 × 300	3
	50 × 100	4

**Table 8:** Summary of study cases.

Aim of Research	Materials	Laboratories	Results	Ref.
Determination of the compressive and flexural strength behavior of unstressed concrete samples embedded in polluted soil	➢ Ordinary Portland cement grade 42.5 ➢ Dimension of cubic samples: 15cm × 15cm × 15cm ➢ Dimension of beams: 15cm × 15cm × 100cm ➢ Concrete mix 1:1.5:3	➢ Compressive and flexural test at curing ages of 28 up to 196 days ➢ Compressive strength of concrete samples exposed to progressive heat in five cycles ➢ Consistency, gravity, soundness, and compressive strength of cement ➢ Determination of moisture, bulk density, and the gravity of aggregates	➢ Reduction in the compressive strength up to 9.47% during the first 28 days ➢ Reduction in the flexural strength up to 34.50% during the first 28 days	[53]
Analyze the influence of organic abattoir waste and disposal hydrocarbon contamination on the durability of concrete	➢ Ordinary Portland cement grade 42.5 ➢ Dimension of cubic samples: 15cm × 15cm × 15cm ➢ Dimension of beams: 15cm × 15cm × 90cm ➢ Steel for beams Ø10mm and Ø8mm	➢ Compressive strengths of samples (every seven days until the 84th day in cubes) ➢ Flexural strength at the age of 84 days ➢ Density of concrete ➢ Physical and chemical properties of contaminated and not contaminated soil	➢ The physical and mechanical properties of the concrete were affected by the presence of soil contaminants ➢ Hydrocarbon contamination had a more significant effect on the load-carrying capacity of concrete	[54]
Determination of the influence of crude oil on the compressive strength of concrete	➢ Dimension of samples: 15cm × 15cm × 15cm ➢ Concrete mix with 0%, 1%, 2%, 3%, 4%, and 5% of contaminated aggregates	➢ Characterization of physical properties of aggregates used to manufacture the concrete. ➢ Concrete Slump Test ➢ Compressive strength at 7, 14, 28, and 56 days	➢ The presence of crude oil in concrete samples significantly decreased the mechanical properties ➢ Increase in percentages of crude oil in the fine aggregate cause higher workability of concrete	[52]
Analyze the mechanical and physical properties behavior of concrete samples	➢ Dimension of samples: Ø50 mm × 100 mm ➢ Dimension of samples: prismatic: 25 mm × 25 mm × 285 mm ➢ ${\mathrm{Na}}_{2}{\mathrm{SO}}_{4\;}$ solution	➢ Compressive strength ➢ Measurement of elastic modulus ➢ Permeability ➢ Expansion behavior ➢ Carbonation depth	➢ Increments in the expansion material of about 0.5% higher than the limit expansion stated in the standards ➢ Compressive strength shows a reduction in the resistance of about 30%	[55]
Comparison of the concentration and intensity distribution of $\mathrm{Ca}{\left(\mathrm{OH}\right)}_{2}{\;\mathrm{and}\;\mathrm{CaCO}}_{3}$ in concrete samples	➢ Dimension of samples: Ø150 mm × 300 mm ➢ Type I ordinary Portland cement ➢ Phenolphthalein indicator	➢ Carbonation depth ➢ Thermalgravimetric analysis (TGA) method ➢ X-ray diffraction analysis tests ➢ Fourier transformation infrared spectroscopy (FTIR) test ➢ pH measurement ➢ Compressive strength	➢ TGA, FTIR, and XRDA test show very similar results in the carbonation depth of about 35 mm up to 16 weeks ➢ Carbonation depth measured by the phenolphthalein test shows a value of 17 mm in the same frame of time	[57]
Analyze the behavior of corrosion in reinforced concrete embedded in soil contaminated with chlorides and sulfates	➢ Dimension of samples: 120 mm × 70 mm × 180 mm ➢ Soil type MH ➢ Portland cement, CPC 30R RS, and CPC 30R ➢ Steel bars of AISI 1018 Carbon Steel and Galvanized Steel, Ø 3/8 and steel bars of UNS S31600	➢ Characterization of concrete mixtures in a fresh state ➢ Initial compressive strength ➢ Measurement of corrosion potential ➢ Physical description of the soil	➢ Concrete samples exposed to soil contamination with NaCl content higher to 2% present the highest probability to suffer from premature corrosion in the steel bars during the first 103 days ➢ Lower Icorr magnitudes in samples made with Portland type V	[58]
Evaluation of the electromechanical behavior of concrete samples embedded in contaminated soil with different percentages of magnesium sulfate $\left({\mathrm{MgSO}}_{4}\right)$	➢ Dimension of samples: 120 mm × 70 mm × 180 mm ➢ Soil type SP ➢ Portland cement, CPC 30R RS, and CPC 30R ➢ Steel bars of AISI 1018 carbon steel and galvanized steel Ø 3/8” and bars of UNS S31600	➢ Measurement of corrosion potential ➢ Measurement of corrosion kinetics	➢ In concentrations between 1% and 2% of $MgSO_{4}\;$ the corrosion resistance varies according to the Portland cement and steels bars type, being higher in concrete made with CPC 30R RS and reinforced with galvanized bars ➢ All concrete samples present a high and moderate level of corrosion during the first 130 days in soils, with 3% of $MgSO_{4}\;$ content	[66]
Evaluation of the corrosion behavior of carbon and stainless steel bars using different concrete mixtures, including the addition of silica fumes and fly ash	➢ Dimension of samples: Ø150mm× 300mm and 120mm × 70mm × 150mm ➢ AISI 1018 carbon steel and AISI 304 stainless steel with Ø 0.95 mm ➢ Concrete mixtures, 100% CPC, 80% CPC, and 20% silica fume, and 80% CPC and 20% fly ash	➢ Measurement of corrosion potential ➢ Characterization of concrete aggregates ➢ Physical and mechanical characterization of fresh and hardened concrete mixtures ➢ Initial compressive strength	➢ Severe corrosion in all concrete samples during the 365 days of exposure ➢ Samples with 20% of fly ash and silica fume addition showed a reduction of around 70% in the kinetic corrosion in comparison with the specimens without mineral additions	[67]

**Table 9:** Comparison of the most popular coatings for concrete elements [25,31,68,69,70,71,72,73,74,75,76].

Type of Coating	Advantages	Disadvantages
**Epoxy resin**	✓ Excellent adhesion properties on different substrates	✗ Poor impact resistance
	✓ High chemical and solvent resistance	✗ Low-temperature resistance
	✓ Control concrete carbonation	✗ Inherent brittleness
	✓ Fluidity in the application due to its low viscosity properties	✗ Inferior weathering resistance
	✓ Good electrical properties	✗ Complex removal procedure
	✓ Excellent anticorrosion performance	✗ Costly maintenance
		✗ Strong toxic fumes
**Bitumen**	✓ Good penetration into the surface due to its fluidity	✗ Its protectiveness can be affected by polymer grade
	✓ When used in pavements, it improves the sticking between different layers and increases the resistance to deformation	✗ It is affected by the temperature in the summer season by making the coating soft
	✓ High water resistance	✗ Difficult to apply to plastic surfaces
	✓ High resistance to mechanical damage	✗ Overheat buildings when it is used to the roof
	✓ High resistance to UV radiation	
**Acrylics**	✓ Highly resistant to variations in temperature	✗ Complex removal procedure
	✓ High impact resistance	✗ Fast drying
	✓ High chemical resistance	✗ Poor water repellent
	✓ User friendly, easy to apply	✗ Low UV radiation resistance
	✓ High fungus resistance	
	✓ Lower cost applications	
	✓ Good adhesion properties	
**Polyurethane resin**	✓ High performance in its mechanical properties such as flexibility, strength, hardness, and stiffness	✗ It is sensitive to humidity
	✓ Control concrete carbonation	✗ Delays the natural breathing capability of concrete
	✓ Long service life	✗ Low weathering resistance
	✓ High resistance to UV radiation	✗ Strong toxic fumes
	✓ Economic maintenance	✗ Less alkali-resistant than epoxy coating
	✓ High hardness and impact resistance	✗ High cost

**Table 10:** Standard procedures for the determination of mechanical and physical properties of coating materials.

Standard Reference	Standard Title	Parts
**PN-EN ISO 2811**	Density determination, paints, and varnishes	Part 1: Pycnometric method (2016)
		Part 2: Immersed body (plummet) method (2011)
		Part 3: Oscillation method (2011)
		Part 4: Pressure cup method (2011)
**PN-EN ISO 2884:2007**	Viscosity determination, paints, and varnishes	Part 1: High shear cone-plate viscometer
		Part 2: Viscometer with disc or ball, fixed speed
**PN-EN ISO 2431:2019**		Part 1: Determination of flow time by use of flow-cups
**PN-EN ISO 2808: 2020**	Measurement of coating thickness, paint, and varnishes	Part 1: Determination of the coating thickness
**PN-EN ISO 2178: 2016**		Part 1: Non-magnetic coatings on a magnetic substrate—magnetic method
**PN-EN ISO 2360: 2017**		Part 1: Amplitude-sensitive eddy-current method
**PN-EN ISO 4624: 2016**	Adhesion of the coating to the substrate, paints, and varnishes	Part 1: Pull of test
**PN-EN ISO 2409: 2013**		Part 1: Cross-cut test
**PN-EN 14891:2017**	Ceramic tiling bonded with adhesives - requirements, test methods, and liquid applied water-impermeable products.	

**Table 11:** Summary of laboratories for coating materials.

Standard Reference Used	Type of Laboratory Performed	Number of Articles
**PN-EN ISO 62:2008**	Water absorption	1
**ASTM C642-97**		2
**PN-EN ISO-527-1,3**	Tensile stress	2
**PN-EN 14891:2012/17**		3
**ZUAT-15/IV.13/2002**	Adhesion	1
**ASTM D4541-17**		2
**ZUAT-15/IV.13/2002**	Resistance to freeze/thaw cycles	1
**PN-EN 1504-2:2006**		2
**PN-EN 14891:2017**		2
**ASTM D562-10**	Viscosity	1
**PN-EN 1504-2:2006**	Ability to cover cracks	2
**EN ISO 9117-1:2009**	Curing time	1
**ASTM D1640**		2
**ASTM C642-97**	Water absorption	2

## Abbreviations

**Abbreviation**	**Meaning**
$\;{\;}^{137}Cs$	Cesium 137
${\;}^{134}Cs$	Cesium 134
**ACO**	Acetoxy group
**AISI**	American Iron and Steel Institute
**ASR**	Alkali–silica reaction
**As/PU**	Asphalt and polyurethane
**ASTM**	American Society for Testing and Materials
**BC**	Before Christ
**BS**	British standard
$CaCO_{3}$	Calcium carbonate
**CEM**	Cement
**CPC**	Calcium phosphate cements
$C_{3}A$	Tricalcium aluminate
$CO_{2}$	Carbon dioxide
$CO_{x}$	Carbon oxides
**DGEBA**	Diglycidyl ether of bisphenol A
**EN**	European standards
**GPa**	Gigapascal
**ISO**	International Organization for Standardization
**ITB**	Building Research Institute
**KOH**	Potassium hydroxide
**LiOH**	Lithium hydroxide
**MgSO4**	Magnesium sulfate
$Na_{2}CO_{3}$	Sodium carbonate
$Na_{2}SO_{4}$	Sodium sulfate
**NaCl**	Sodium chloride
**NaOH**	Sodium hydroxide
**NCO**	Isocyanate chemical group
$NO_{x}$	Nitrogen oxide
**OH**	Hydroxide
**pH**	Potential of hydrogen
**PN**	Polish standards
**PVC**	Polyvinyl chloride
**RC**	Reinforced concrete
**SEM**	Scanning electron microscopy
**SER**	Solid epoxy resins
$SO_{x}$	Sulfur oxide
**TGA**	Thermalgravimetric analysis
**UNS**	Unified number system
$UO_{x}$	Uranium oxide
**UV**	Ultraviolet
**W/C**	Water/cement
**XRD**	X-ray diffraction
**ZUAT**	Recommendations of the Technical Approval Provision

## References

[1] Popov B.N. (2015). Corrosion of Structural Concrete. Corros. Eng..

[2] Safiuddin M. (2017). Concrete Damage in Field Conditions and Protective Sealer and Coating Systems. Coatings.

[3] Vaysburd A., Emmons P. (2000). How to make today’s repairs durable for tomorrow—corrosion protection in concrete repair. Constr. Build. Mater..

[4] Shi X., Xie N., Fortune K., Gong J. (2012). Durability of steel reinforced concrete in chloride environments: An overview. Constr. Build. Mater..

[5] Owsiak Z. (2010). Testing alkali—Reactivity of selected concrete aggregates. J. Civ. Eng. Manag..

[6] Figueira R.B., Sousa R., Coelho L., Azenha M., de Almeida J.M., Jorge P.A.S., Silva C.J.R. (2019). Alkali-silica reaction in concrete: Mechanisms, mitigation and test methods. Constr. Build. Mater..

[7] Portland Cement Association Types and Causes of Concrete Deterioration. Portl. Cem. Assoc. Concr. Inf. PCA R.D. Se 2002, 5420 Old Orchard Road, 1–16. www.cement:docs/default-source/fc_concrete_technology/durability/is536-types-and-causes-of-concrete-deterioration.pdf?sfvrsn=4&sfvrsn=4.

[8] Wang K. Carbonate Aggregate in Concrete. http://www.lrrb:pdf/201514.pdf.

[9] Wang C., Chen F. (2019). Durability of Polypropylene Fiber Concrete Exposed to Freeze-Thaw Cycles with Deicing Salts.

[10] Enshassi A., Kochendoerfer B., Rizq E. (2014). Evaluación de los impactos medioambientales de los proyectos de construcción. Rev. Ing. Construcción.

[11] Zolfagharian S., Nourbakhsh M., Irizarry J., Ressang A., Gheisari M. Environmental impacts assessment on construction sites. Proceedings of the Construction Research Congress 2012: Construction Challenges in a Flat World, EE. UU.

[12] Bonić Z., Ćurčć G.T., Davidovič N., Savič J. (2015). Damage of concrete and reinforcement of reinforced-concrete foundations caused by environmental effects. Procedia Eng..

[13] Popov B.N. (2015). High-Temperature Corrosion. Corros. Eng. Elsevier.

[14] Stambaugh N.D., Bergman T.L., Srubar W.V. (2018). Numerical service-life modeling of chloride-induced corrosion in recycled-aggregate concrete. Constr. Build. Mater..

[15] Xia J., Shen J., Li T., Jin W.-L. (2021). Corrosion prediction models for steel bars in chloride-contaminated concrete: A review. Mag. Concr. Res..

[16] Yin G.J., Zuo X.B., Tang Y.J., Ayinde O., Wang J.L. (2017). Numerical simulation on time-dependent mechanical behavior of concrete under coupled axial loading and sulfate attack. Ocean Eng..

[17] Silva M.A.G., Cunha M.P., Pinho-Ramos A., da Fonseca B.S., Pinho F.F.S. (2017). Accelerated action of external sulfate and chloride to study corrosion of tensile steel in reinforced concrete. Mater. Constr..

[18] Pernicová R., Dobiáš D. (2017). Resistance of surface layers of concrete against aggressive environment. Key Eng. Mater..

[19] Wei J., Liu J., Bai Y., Song Z., Feng Q., Lu Y., Sun S. (2018). Effect on the resistance of concrete acid corrosion in superficial soil layers. Adv. Civ. Eng..

[20] Kozubal J., Wyjadłowski M., Steshenko D. (2019). Probabilistic analysis of a concrete column in an aggressive soil environment. PLoS ONE.

[21] Li L., Li J., Yang C. (2019). Theoretical approach for prediction of service life of RC pipe piles with original incomplete cracks in chloride-contaminated soils, Constr. Build. Mater..

[22] Kim J.-H.J., Lim Y.M., Won J.P., Park H.G. (2010). Fire resistant behavior of newly developed bottom-ash-based cementitious coating applied concrete tunnel lining under RABT fire loading. Constr. Build. Mater..

[23] Fattuni N.I., Hughes B.P. (1983). Effect of acid attack on concrete with different admixtures or protective coatings. Cem. Concr. Res..

[24] Taylor S.R. (2001). Coatings for Corrosion Protection: Inorganic. Encycl. Mater. Sci. Technol..

[25] Taylor S.R. (2001). Coatings for Corrosion Protection: Organic. Encycl. Mater. Sci. Technol. Elsevier.

[26] Guma T.N., Aku S.Y., Yawas D.S., Dauda M. (2015). Bitumen in Coating Corrosion Protection of Steel-The Position and Prognosis of Nigerian Bitumen. Am. J. Eng. Res..

[27] Raghav P.K., Agarwal N., Saini M. (2016). Edible Coating of Fruits and Vegetables: A Review View Project. www.researchgate.net/publication/331298687.

[28] Singh A.K., Bhadauria A.S., Kumar P., Bera H., Saha S. (2019). Bioactive and drug-delivery potentials of polysaccharides and their derivatives. Polysaccharide Carriers for Drug Delivery.

[29] Popov B.N. (2015). Organic Coatings. Corros. Eng. Elsevier.

[30] Pham H.Q., Marks M.J. (2005). Epoxy Resins, Assesments of Potential BPA Emissions. Construction Research Congress 2012: Construction Challenges in a Flat World.

[31] Chattopadhyay D.K., Raju K.V.S.N. (2007). Structural engineering of polyurethane coatings for high performance applications. Prog. Polym. Sci..

[32] Americus (1978). Coatings update: Chlorinated rubber technology. Pigment Resin Technol..

[33] Ibrahim M., Al-Gahtani A.S., Maslehuddin M., Dakhil F.H. (1999). Use of surface treatment materials to improve concrete durability. J. Mater. Civ. Eng..

[34] Satoshi S. (2005). Solution Casting Method. U.S. Patent Application.

[35] Myers R.R. (1981). History of Coatings Science and Technology. J. Macromol. Sci. Part A. Chem..

[36] Edwards K.N., Mislang H.B. (2000). History of coatings. Appl. Polym. Sci. 21st Century Elsevier.

[37] Soucek M., Johansson M.K.G. (2012). Alkyds for the 21st century. Prog. Org. Coatings.

[38] Pilcher G.R., The ChemQuest Group (2019). Entering the Second Decade of the 21st Century: The State of the U.S. Paint and Coatings Industry. CoatingsTech.

[39] Merachtsaki D., Tsardaka E.-C., Tsampali E., Simeonidis K., Anastasiou E., Yiannoulakis H., Zouboulis A. (2020). Study of Corrosion Protection of Concrete in Sewage Systems with Magnesium Hydroxide Coatings. Environ. Sci. Proc..

[40] Aguirre-Guerrero A.M., de Gutiérrez R.M. (2021). Alkali-activated protective coatings for reinforced concrete exposed to chlorides. Constr. Build. Mater..

[41] Sakr M.R., Bassuoni M.T., Taha M.R. (2019). Effect of coatings on concrete resistance to physical salt attack. ACI Mater. J..

[42] Ferenhof H.A., Fernandes R.F. Desmistificando a Revisão de Literatura Como Base Para Redação cientíFica: Método SSF, n.d. www.researchgate.net/publication/325070845.

[43] Lelieveld J., Evans J.S., Fnais M., Giannadaki D., Pozzer A. (2015). The contribution of outdoor air pollution sources to premature mortality on a global scale. Nature.

[44] Saha J.K., Selladurai R., Kundu S., Patra A.K. (1989). Chapter 9 Water, Agriculture, Soil and Environmental. Tech. Instrum. Anal. Chem..

[45] Mirsal I.A. (2004). Soil Pollution. Origin, Monitoring and Remediation.

[46] Pallise J. (2014). Impactos ambientales de la producción de electricidad. Asoc. Prod. Energías Renov..

[47] Zwolak A., Sarzyńska M., Szpyrka E., Stawarczyk K. (2019). Sources of Soil Pollution by Heavy Metals and Their Accumulation in Vegetables: A Review. Water Air Soil Pollut..

[48] Rodríguez N., McLaughlin F.M., Pennock D. (2018). Soil Pollution: A Hidden Reality. www.fao.org/.

[49] Owa F.D. (2013). Water pollution: Sources, effects, control and management. Mediterr. J. Soc. Sci..

[50] Haufe J., Vollpracht A. (2019). Tensile strength of concrete exposed to sulfate attack. Cem. Concr. Res..

[51] Meng C., Li W., Cai L., Shi X., Jiang C. (2020). Experimental research on durability of high-performance synthetic fibers reinforced concrete: Resistance to sulfate attack and freezing-thawing. Constr. Build. Mater..

[52] Osuji S., Nwankwo E. (2015). Effect of Crude Oil Contamination on the Compressive Strength of Concrete. Niger. J. Technol..

[53] Adewuyi A.P., Olaniyi O.A., Olafusi O.S., Fawumi A.S. (2015). Compressive and Flexural Behaviour of Unstressed Concrete Substructure in Cassava Effluent Contaminated Soils. Open J. Civ. Eng..

[54] Adewuyi A. (2017). Strength and durability assessment of concrete substructure in organic and hydrocarbon polluted soil. Int. J. Mod. Res. Eng. Technol..

[55] Yu X.T., Chen D., Feng J.R., Zhang Y., di Liao Y. (2018). Behavior of mortar exposed to different exposure conditions of sulfate attack. Ocean Eng..

[56] Jedidi M., Benjeddou O. (2018). Chemical causes of concrete degradation. MOJ Civ. Eng..

[57] Chang C.-F., Chen J.-W. (2006). The experimental investigation of concrete carbonation depth. Cem. Concr. Res..

[58] Baltazar-Zamora M.A., Mendoza-Rangel J.M., Croche R., Gaona-Tiburcio C., Hernández C., López L., Olguín F., Almeraya-Calderón F. (2019). Corrosion Behavior of Galvanized Steel Embedded in Concrete Exposed to Soil Type MH Contaminated With Chlorides. Front. Mater..

[59] Melchers R.E., Li C.Q. (2009). Reinforcement corrosion initiation and activation times in concrete structures exposed to severe marine environments. Cem. Concr. Res..

[60] Ramli M., Kwan W.H., Abas N.F. (2013). Strength and durability of coconut-fiber-reinforced concrete in aggressive environments. Constr. Build. Mater..

[61] Zhong R., Wille K. (2018). Deterioration of residential concrete foundations: The role of pyrrhotite-bearing aggregate. Cem. Concr. Compos..

[62] Oliveira I., Cavalaro S.H.P., Aguado A. (2014). Evolution of pyrrhotite oxidation in aggregates for concrete. Mater. Construcción.

[63] Tagnit-Hamou A., Saric-Coric M., Rivard P. (2005). Internal deterioration of concrete by the oxidation of pyrrhotitic aggregates. Cem. Concr. Res..

[64] Yoshida N. (2019). Sulfate attack on residential concrete foundations in Japan. J. Sustain. Cem. Mater..

[65] Tulliani J.M., Montanaro L., Negro A., Collepardi M. (2002). Sulfate attack of concrete building foundations induced by sewage waters. Cem. Concr. Res..

[66] Santiago-Hurtado G. (2016). Electrochemical evaluation of reinforcement concrete exposed to soil type SP contaminated with sulphates. Int. J. Electrochem. Sci..

[67] Baltazar-Zamora M.A., Bastidas D.M., Santiago-Hurtado G., Mendoza-Rangel J.M., Gaona-Tiburcio C., Bastidas J.M., Almeraya-Calderón F. (2019). Effect of Silica Fume and Fly Ash Admixtures on the Corrosion Behavior of AISI 304 Embedded in Concrete Exposed in 3.5% NaCl Solution. Materials.

[68] Hu H., He Y., Long Z., Zhan Y. (2017). Synergistic effect of functional carbon nanotubes and graphene oxide on the anti-corrosion performance of epoxy coating. Polym. Adv. Technol..

[69] Patil R. (2016). Waterproofing: Types, Advantages & Disadvantages. constructionor.com/waterproofing/.

[70] Rabiot D., Morizur M.F. (1996). Polymer-modified bitumen emulsions an advantage for the various road applications. Eurasphalt Eurobitume Congr..

[71] Paliukaite M., Vorobjovas V., Bulevičius M., Andrejevas V. (2016). Evaluation of Different Test Methods for Bitumen Adhesion Properties. Transp. Res. Procedia.

[72] Nguyen T.N.L., Do T.V., Nguyen T.V., Dao P.H., Trinh V.T., Mac V.P., Nguyen A.H., Dinh D.A., Nguyen T.A., Vo T.K.A. (2019). Antimicrobial activity of acrylic polyurethane/Fe3O4-Ag nanocomposite coating. Prog. Org. Coatings.

[73] Alessandrini G., Aglietto M., Castelvetro V., Ciardelli F., Peruzzi R., Toniolo L. (2000). Comparative evaluation of fluorinated and unfluorinated acrylic copolymers as water-repellent coating materials for stone. J. Appl. Polym. Sci..

[74] Samimi A., Zarinabadi S. (2012). Application Polyurethane as Coating in Oil and Gas Pipelines Calculation of Corrosion in Oil and Gas Refinery with EOR Method View project Application Polyurethane as Coating in Oil and Gas Pipelines. Int. J. Sci. Eng. Investig..

[75] (2019). Alchimica, Advantages and Disadvantages of Polyurethane Waterproofing Materials. alchimica.com.ua/en/2019/05/13/advantages-and-disadvantages-of-polyurethane-waterproofing-materials/.

[76] Kanimozhi K., Prabunathan P., Selvaraj V., Alagar M. (2016). Bio-based silica-reinforced caprolactam-toughened epoxy nanocomposites. High Perform. Polym..

